# Supplementary material for: The simplified hybrid model based on BP to predict the reference crop evapotranspiration in Southwest China
Source: PLoS One. 2022 Jun 13;17(6):e0269746. doi: 10.1371/journal.pone.0269746 (PMC9191727; doi:10.1371/journal.pone.0269746)
Supplement: S1 Appendix — (PDF) [file pone.0269746.s001.pdf]

S1 Appendix. Raw results of factor importance.

Station	Ta	Tmax	Tmin	Wind	RH	Ra	n
Liuzhou	0.005132	0.647699	0.00235	0.016366	0.012747	0.060119	0.255587
Tongren	0.002958	0.649146	0.000705	0.012734	0.014083	0.076752	0.243623
Baise	0.003012	0.628673	0.001431	0.008702	0.009905	0.070313	0.277965
Nanning	0.003664	0.570595	0.00178	0.020499	0.031266	0.148894	0.223302
Baoshan	0.001525	0.638832	0.000731	0.008767	0.023008	0.051474	0.275663
Yuxi	0.002462	0.652662	0.000606	0.007481	0.010589	0.080775	0.245425
Mengzi	0.009368	0.680005	0.002936	0.010181	0.016918	0.037573	0.243019
Barkam	0.002906	0.696314	0.001081	0.006703	0.007204	0.046246	0.239546
Yaan	0.006521	0.405449	0.002984	0.01499	0.14611	0.166509	0.257437
Bazhong	0.005919	0.501331	0.001985	0.016066	0.145488	0.074264	0.254948
Kaili	0.004748	0.548573	0.002464	0.029026	0.067254	0.050894	0.297042
Liangping	0.004615	0.704773	0.000508	0.003882	0.010051	0.059804	0.216365
