# Supplementary material for: The simplified hybrid model based on BP to predict the reference crop evapotranspiration in Southwest China
Source: PLoS One. 2022 Jun 13;17(6):e0269746. doi: 10.1371/journal.pone.0269746 (PMC9191727; doi:10.1371/journal.pone.0269746)
Supplement: S2 Appendix — (PDF) [file pone.0269746.s002.pdf]

S2 Appendix. Raw results of the ET_0 model.

Site	BP model			
	RMSE	R^2	MAE	NSE
Liuzhou	0.84391	0.930042	0.627621	0.663126
Tongren	0.815794	0.758862	0.65169	0.657567
Baise	0.590088	0.851364	0.482489	0.833385
Nanning	0.844834	0.603967	0.669941	0.599782
Baoshan	0.868646	0.499561	0.686321	0.404576
Yuxi	0.915939	0.582323	0.72372	0.525209
Mengzi	0.865793	0.642583	0.692805	0.562979
Barkam	0.895161	0.546051	0.759065	0.399528
Yaan	0.58934	0.840651	0.44666	0.813172
Bazhong	0.487675	0.910689	0.386194	0.905273
Kaili	0.787759	0.897719	0.575032	0.706086
Liangping	0.844599	0.788263	0.616419	0.716889
Liuzhou	0.84391	0.930042	0.627621	0.663126
Tongren	0.815794	0.758862	0.65169	0.657567
Baise	0.590088	0.851364	0.482489	0.833385
Nanning	0.844834	0.603967	0.669941	0.599782

Site	ACO-BP			
	RMSE	R^2	MAE	NSE
Liuzhou	0.537436	0.96223	0.396708	0.863375
Tongren	0.721625	0.953785	0.484405	0.73206
Baise	0.600936	0.82905	0.483544	0.827203
Nanning	0.526858	0.863971	0.44255	0.844353
Baoshan	0.479058	0.932844	0.354185	0.818901
Yuxi	0.421726	0.932092	0.287247	0.899346
Mengzi	0.472948	0.923176	0.387369	0.869593
Barkam	0.466832	0.887243	0.373557	0.836691
Yaan	1.097391	0.681795	0.945245	0.352213
Bazhong	1.13076	0.803918	0.775796	0.490725
Kaili	0.743715	0.919674	0.658541	0.738033
Liangping	0.754864	0.851321	0.623714	0.773852
Liuzhou	0.537436	0.96223	0.396708	0.863375
Tongren	0.721625	0.953785	0.484405	0.73206
Baise	0.600936	0.82905	0.483544	0.827203
Nanning	0.526858	0.863971	0.44255	0.844353

Site	CSO-BP			
	RMSE	R^2	MAE	NSE
Liuzhou	0.252095	0.972317	0.185766	0.969939
Tongren	0.23022	0.974688	0.151791	0.972729
Baise	0.281698	0.963944	0.202639	0.96203
Nanning	0.205204	0.976683	0.152849	0.976388
Baoshan	0.281103	0.949769	0.189358	0.937645
Yuxi	0.376894	0.935315	0.248003	0.919609
Mengzi	0.347891	0.932247	0.260728	0.92944

Barkam	0.259177	0.951035	0.186462	0.949663
Yaan	0.232966	0.971012	0.170441	0.970806
Bazhong	0.232838	0.97901	0.144782	0.978407
Kaili	0.273457	0.965056	0.194036	0.964583
Liangping	0.199734	0.984294	0.139508	0.984167
Liuzhou	0.252095	0.972317	0.185766	0.969939
Tongren	0.23022	0.974688	0.151791	0.972729
Baise	0.281698	0.963944	0.202639	0.96203
Nanning	0.205204	0.976683	0.152849	0.976388

Site	CS-BP			
	RMSE	R^2	MAE	NSE
Liuzhou	0.25754	0.971249	0.189933	0.968626
Tongren	0.23365	0.974282	0.158196	0.97191
Baise	0.292427	0.961055	0.211825	0.959082
Nanning	0.208656	0.9757	0.154902	0.975587
Baoshan	0.292269	0.947003	0.202818	0.932593
Yuxi	0.386718	0.930467	0.254835	0.915363
Mengzi	0.347275	0.932207	0.264613	0.929689
Barkam	0.265515	0.948511	0.193681	0.947171
Yaan	0.24041	0.968965	0.179387	0.96891
Bazhong	0.235269	0.978619	0.149102	0.977953
Kaili	0.274531	0.964758	0.196431	0.964304
Liangping	0.215335	0.981903	0.157867	0.981597
Liuzhou	0.25754	0.971249	0.189933	0.968626
Tongren	0.23365	0.974282	0.158196	0.97191
Baise	0.292427	0.961055	0.211825	0.959082
Nanning	0.208656	0.9757	0.154902	0.975587
