# Supplementary material for: The simplified hybrid model based on BP to predict the reference crop evapotranspiration in Southwest China
Source: PLoS One. 2022 Jun 13;17(6):e0269746. doi: 10.1371/journal.pone.0269746 (PMC9191727; doi:10.1371/journal.pone.0269746)
Supplement: S3 Appendix — (PDF) [file pone.0269746.s003.pdf]

S3 Appendix. The pseudocode of the proposed model.

```
Input:
Population size sizepop
Maximum number of iterations maxiter
The number of input parameter inputnum
The number of output parameter outputnum
BP structure net
Number of samples sample
Range of genetic changes popmax
Fitness function fun
Upper and lower limits of speed Vmax, Vmin
Function CSO_ITR(maxiter, sizepop):
    Initialize:
    for i in 1:sizepop do
            pop[i,:] ← popmax*rands(1, sample) //generate random number in [-5,5]
            V[i,:] ← rands(1,sample)
      fitness[i] ← fun(pop[i,:],inputnum,hiddennum,outputnum,net)
    end
        fitnessgbest ← record the current individual extreme value
    fitnesszbest ← record the current population extreme value
  Iterative Refinement:
      for i in 1:maxiter do
        for j in 1:sizepop do
            //update velocity
                V[j,:] ← V[j,:] + c1*rand*(gbest[j,:]- pop[j,:]) + c2*rand*(zbest – pop[j,:])
            Limit V to [Vmin, Vmax]
              // update population
            pop[j,:] ← pop[j,:]+0.2*V[j,:]
            Limit pop to [popmin, popmax]
            Adaptive variation
            Update Fitness value
        end
        for k in 1:sizepop do
                  //Individually optimal update
            if   fitness[k] < fitnessgbest[k] then
                   gbest[k,:] ← pop[k,:]
                fitnessgbest[k] ← fitness[k]
            end
            // group optimal update
        if fitness[k]<fitnesszbest[k] then
            zbest ← pop[k,:]
        fitnesszbest=fitness[k]
        end
```

end

// The optimal value zbest of the swarm algorithm is used as the initial weight of the network BP

Train(net)

return net(test_data)
