# Supplementary material for: The simplified hybrid model based on BP to predict the reference crop evapotranspiration in Southwest China
Source: PLoS One. 2022 Jun 13;17(6):e0269746. doi: 10.1371/journal.pone.0269746 (PMC9191727; doi:10.1371/journal.pone.0269746)
Supplement: Attachment — Submitted filename: Response to Reviewers.docx [file pone.0269746.s004.docx]

Dear editor,

Thank you for your consideration of this manuscript. We appreciate all valuable comments, and all revises are noted in the file named as “Revised Manuscript with Track Changes”. The reply to the request are as follow：

1. We rechecked and corrected the format of the manuscript.

2. The corresponding author verified and updated the ORCID ID （0000-0003-2018-9595）。

3. We have deleted Figure 1 from the manuscript.

4. We have added citations to Figs 2 and 3 in the paper.

Sincerely,
Zhenhua Zhao, Guohua Feng, Jing Zhang

**Response to Comments**

Reviewer #1

Question1. Lines 38-40 is better to be rewritten in order to be more comprehensive.

Response：Thank you for your advice. As you suggested, the sentence has been revised. “However, due to the uneven distribution and inconsistent scale of meteorological stations, it is difficult to obtain all meteorological parameters used to calculate ET_O_, which makes it difficult to accurately calculate ET_O_.”. (Line45-Line46 in “Revised Manuscript with Track Changes”)

Question2. The references need to be rewritten in the journal’s style. In some references are missing the year of the publication.

Response：We revised the reference format according to the requirements of the journal and added the missing publication year.

Reviewer #2

Question1. The manuscript has problems in the use of English and several parts are difficult to understand. There are some format issues like some spaces are missing before brackets in the main text and in the References as well.

Response：Thank you for your valuable suggestions. We carefully reexamined our manuscript, found some language errors, and revised them. The spaces before the brackets have been added in the main text and in the references.

Question2.38–40: “However, due to the uneven distribution and inconsistent scale of meteorological stations, which makes it difficult to accurately calculate ETO.” You could rephrase that.

Response：The sentence has been revised to “However, due to the uneven distribution and inconsistent scale of meteorological stations, it is difficult to obtain all meteorological parameters used to calculate ET_O_, which makes it difficult to accurately calculate ET_O_.”. (Line45-Line46 in “Revised Manuscript with Track Changes”)

Question3. 59 – 62: I think some articles are missing. You could rephrase that.

Response：We added the missing sentence "and many scholars use optimization algorithms to improve the accuracy of models in recent years.", which made the content of the article more coherent. (Line69-Line71)

Question4.63 – 64: “A more efficient prediction model with better adaptability is obtained.” It is not clear what you are referring to.

Response：We have modified sentences with ambiguous semantics. “Fang et al. ^[18]^ used the fruit fly optimization algorithm (FOA) to optimize the ET_O_ constructed by generalized regression neural networks (GRNN) algorithm, and obtained a more efficient and adaptive prediction model.” (Line71-Line74)

Question5. 67:“Comprehensive meteorological parameters are difficult to obtain”. You should rephrase that.

Response：The description in the manuscript has been changed to " There are many difficulties in the actual collection of meteorological data, which makes it difficult to obtain all meteorological parameters used to calculate ET_O_,". (Line78-Line80)

Question6. 69 – 71: “determine the dominant factors of ETO model based on path analysis theory, or use principal component analysis and factor analysis to select key determinants.” You could rephrase that.

Response：The description in the manuscript has been changed to " determine the dominant factors of ET_O_ model based on path analysis theory, principal component analysis and factor analysis." (Line83-Line84)

Question7. 103: I see 12 stations in the analysis.

Response：The number of sites has been modified. (Line116)

Question8. 118: " is the solar radiation of surface net", net solar radiation

Response：The description has been modified to "net solar radiation". (Line132)

Question9. Please correct the format of the units’ titles 2.3.1, 2.3.2 and 2.3.2.

Response：The format of the title has been modified according to the requirements of the journal.

Question10. Bold fonts are detected in cases where they shouldn't be (numbering of eq. (17 -21)).

Response：The wrong font has been modified.

Question11. There is inconsistency in the symbolism of solar radiation in eq. (1) and the rest of the manuscript.

Response：The symbolism of solar radiation in eq. (1) is net solar radiation (Rn) and the rest of the manuscript is extraterrestrial solar radiation (Ra). We have concretized the definition of Ra in the paper. (Line 125)

Question12. 299: “from the figure that the accuracy”. Table instead of figure.

Response：The word "figure" has been replaced with "table". (Line 323)

Question13. 318 – 319: “In contrast, CSO-BP has the best performance in constructing ETO prediction model”. This sentence is not clear.

Response：The description in the manuscript has been changed to “Among all ET_O_ models, CSO-BP model has the best accuracy advantage in estimating ET_O_ at each site.” (Line342-Line344)

We tried our best to improve the manuscript. Changes were marked in red in revised paper (Revised Manuscript with Track Changes). We appreciate for Editors/Reviewers’ warm work earnestly, and hope that the correction will meet with positive responses. Once again, many special thanks for your good comments and suggestions!
