# Supplementary material for: The simplified hybrid model based on BP to predict the reference crop evapotranspiration in Southwest China
Source: PLoS One. 2022 Jun 13;17(6):e0269746. doi: 10.1371/journal.pone.0269746 (PMC9191727; doi:10.1371/journal.pone.0269746)
Supplement: Attachment — Submitted filename: Response to Reviewers.docx [file pone.0269746.s005.docx]

Dear editor,

Thank you for your consideration of this manuscript. We appreciate all valuable comments. We have uploaded our figure files to the Preflight Analysis and Conversion Engine (PACE) digital diagnostic tool, and resubmitted the figures in the correct format. Each comment is responded below, and all revises are noted in the file named as “Revised Manuscript with Track Changes”.

**Response to Comments**

Question1. Add the condition of compared methods.

Response: We thank the editor for the constructive comments. We supplemented the parameter conditions of compared methods in the manuscript: (1) BP algorithm: training times (net.trainParam.epochs=1000), learning rate (net.trainParam.lr=0.01), Minimum error of training goal (net.trainParam.goal=0.00001), momentum factor (net.trainParam.mc=0.01); (2) ACO algorithm: population size (popsize=10), maximum Generation (maxgen=50), pheromone polatility (rou=0.9), transition probabilities constant (p0=0.2); (3) CSO algorithm: population size(popsize=10), maximum iterations(npop=50), mixture ratio (MR=0.3); (4) CS algorithm: initial population size (PopulationSize_Data=30), probability of cuckoo eggs being found (pa = 0.25), step control amount (cs_alpha=1.0).” (Line143-146, Line 192-194, Line 214-215, Line 254-256)

Question2. Add the pseudo code of the suggested approach.

Response: We supplement the pseudo code of the recommendation model (CSO-BP) in the supporting file. The pseudocode of the proposed model is provided in **S3 Appendix.** (Line422-423)

“**Input:**

Population size *sizepop*

Maximum number of iterations *maxiter*

The number of input parameter *inputnum*

The number of output parameter *outputnum*

BP structure *net*

Number of samples *sample*

Range of genetic changes *popmax*

Fitness function *fun*

Upper and lower limits of speed *Vmax, Vmin*

**Function** CSO_ITR(*maxiter, sizepop*):

**Initialize:**

**for** i in 1:*sizepop* **do**

pop[i,:] ⬅*popmax**rands(1, *sample*) //generate random number in [-5,5]

V[i,:] ⬅rands(1,*sample*)

fitness[i]⬅*fun*(pop[i,:],*inputnum*,*hiddennum*,*outputnum*,*net*)

**end**

*fitnessgbest* ⬅record the current individual extreme value

*fitnesszbest*⬅record the current population extreme value

**Iterative Refinement:**

**for** i in 1:*maxiter* **do**

**for** j in 1:*sizepop* **do**

**//**update velocity

V[j,:]⬅V[j,:] + c1*rand*(gbest[j,:]- pop[j,:]) + c2*rand*(zbest – pop[j,:])

Limit V to [*Vmin, Vmax*]

// update population

pop[j,:] ⬅pop[j,:]+0.2*V[j,:]

Limit pop to [*popmin, popmax*]

Adaptive variation

Update Fitness value

**end**

**for** k in 1:*sizepop* **do**

//Individually optimal update

**if** fitness[k] < fitnessgbest[k] **then**

gbest[k,:] ⬅pop[k,:]

fitnessgbest[k] ⬅fitness[k]

**end**

// group optimal update

**if** fitness[k]<fitnesszbest[k] **then**

zbest⬅pop[k,:]

fitnesszbest=fitness[k]

**end**

**end**

// The optimal value zbest of the swarm algorithm is used as the initial weight of the network BP

Train(net)

**return** net(test_data)”

Question3. To give a better direction for readers add some remarks about potential improvement by the use of type-3 fuzzy logic systems and new learning methods.

Response: In further research, the study scale could serve as a potential improvement. We have supplemented the description in the manuscript. “In future research, we will combine satellite data to conduct a global-scale ET_O_ model applicability study on the hybrid model.” (Line 402-404)

”
